# Genetic etiology study of the non-syndromic deafness in Chinese Hans by targeted next-generation sequencing

**DOI:** 10.1186/1750-1172-8-85

**Published:** 2013-06-14

**Authors:** Tao Yang, Xiaoming Wei, Yongchuan Chai, Lei Li, Hao Wu

**Affiliations:** 1 Department of Otolaryngology–Head and Neck Surgery, Xinhua Hospital, Shanghai Jiaotong University School of medicine, Shanghai, China; 2 Ear Institute, Shanghai Jiaotong University School of medicine, Shanghai, China; 3 BGI-Shenzhen, Shenzhen, China

**Keywords:** Deafness, Non-syndromic, Genetic etiology, Targeted next-generation sequencing

## Abstract

**Background:**

Although over 60 non-syndromic deafness genes have been identified to date, the etiologic contribution of most deafness genes remained elusive. In this study, we addressed this issue by targeted next-generation sequencing of a large cohort of non-syndromic deaf probands.

**Methods:**

Probands with mutations in commonly screened deafness genes *GJB2*, *SLC26A4* and *MT-RNR1* were pre-excluded by Sanger sequencing. The remaining 125 deaf probands proceeded through targeted exon capturing of 79 known deafness genes and Illumina HiSeq2000 sequencing.

**Results:**

Bi-allelic mutations in 15 less commonly screened deafness genes were identified in 28 deaf probands, with mutations in *MYO15A*, *GPR98*, *TMC1*, *USH2A* and *PCDH15* being relatively more frequent (≥3 probands each). Dominant mutations in *MYO6*, *TECTA*, *POU4F3* and *COCH* were identified in 4 deaf families. A mitochondrial *MTTS1* mutation was identified in one maternally inherited deaf family. No pathogenic mutations were identified in three dominant deaf families and two consanguineous families.

**Conclusions:**

Mutations in the less commonly screened deafness genes were heterogeneous and contributed to a significant percentage (17.4%) of causes for non-syndromic deafness. Targeted next-generation sequencing provided a comprehensive and efficient diagnosis for known deafness genes. Complementary to linkage analysis or whole-exome sequencing of deaf families, pre-exclusion of known deafness genes by this strategy may facilitate the discovery of novel deafness genes.

## Background

Hearing impairment, or deafness in its most severe form, is the most common sensory disorder in humans. One in a thousand children develops congenital or prelingual deafness. Another one in a thousand children becomes deaf or severely hearing impaired before adulthood [1]. It was estimated that at least 50% to 60% of childhood hearing impairment was caused by genetic factors [2]. Among them, approximately 70% of cases are non-syndromic, meaning the hearing impairment is the only distinctive clinical feature, and the remaining 30% are syndromic with other abnormalities [3].

Identifying the genetic basis of deafness provides crucial information for diagnosis, intervention and treatment of the disease. The causes, however, are extremely heterogeneous. To date, mutations in 64 genes have been found to be associated with non-syndromic deafness (Hereditary Hearing Loss Homepage, http://webh01.ua.ac.be/hhh/). Most of those genes were originally identified through the linkage analysis of single or multiple deaf families. Expanded screening in large cohorts revealed that mutations in three genes, *GJB2*, *SLC26A4* and *MT-RNR1*, were commonly found in deaf patients. In Chinese Hans, for example, bi-allelic *GJB2* mutations were reported in 19.1% of patients with non-syndromic deafness, followed by bi-allelic *SLC26A4* mutations in 12.1% and the mitochondrial A1555G mutation of *MT-RNR* in 1.6% [4-6]. These genes have been routinely screened during genetic testing and counseling of deafness.

On the other side, the causes remained unknown for majority of deaf individuals. The etiologic contribution of the less commonly screened deafness genes, in particular, has yet to be investigated systematically. Due to the large number and presumably low mutation frequencies of those genes, it would be highly expensive and time-consuming to address this issue by the conventional gene-by-gene Sanger sequencing. This limitation, however, may be overcome by the targeted next-generation sequencing (NGS) technologies. With genomic capturing of a wide array of known deafness genes, it is possible to screen those genes in high throughput by NGS. The feasibility of this strategy has been confirmed in principle in several pilot studies [7-9]. In this study, we performed a comprehensive mutations screening of 79 deafness genes in a large cohort of non-syndromic deaf probands using the targeted NGS. Our results provided a preliminary overview of the genetic etiology of non-syndromic deafness in Chinese Hans.

## Methods

### Recruitment of the subjects

Non-syndromic deaf probands were recruited from deaf patients seeking genetic testing and counseling in Xinhua Hospital, Shanghai, China. Family history and clinical questionnaire was obtained from all subjects or their parents. Physical and neurological examination was performed with special attention to renal, electrocardiac and ophthalmologic differences to exclude those with syndromic deafness. A comprehensive auditory evaluation was performed including otoscope examination, tympanometry, pure-tone audiometry (PTA), auditory brainstem response (ABR) in subjects with very young age, and in some cases computed tomography (CT) of the inner ear. The onset of hearing impairment was categorized as prelingual/early (≤ 6 years) and late (> 6 years). PTA was calculated as the average of the hearing level at 0.5, 1.0, 2.0 and 4.0 KHz for the better ear. The severity of hearing impairment was defined as mild (20~40 dB), moderate (41–70 dB), severe (71~95 dB) and profound (>95 dB). To enroll in this study, all probands should have bilateral, permanent sensorineural hearing impairment. The hearing level of the prelingual or early-onset deaf probands should exceed 70 dB. For the late-onset, progressive probands, their hearing level should exceed 40 dB at the age of 35 years. All subjects or their parents gave written, informed consent to participate in this study. This study was approved by the Ethics Committee of the Shanghai Jiaotong University School of Medicine, Xinhua Hospital.

### Pre-exclusion of mutations in common deafness genes

All deaf probands (n=190) were pre-screened against common deafness genes *GJB2*, *SLC26A4* and *MT-RNR1* by PCR amplification and bidirectional sequencing as previously described [4-6]. A total of 125 deaf probands were void of mutations in the three common deafness genes and proceeded through the targeted exon capturing and NGS.

### Targeted genomic capturing and next-generation sequencing

A customized capture array (NimbleGen, Roche) was designed to capture all exons, splicing sites and immediate flanking intron sequences of 79 deafness genes (Additional file 1: Table S1) as previously described [10]. Genomic DNA were extracted from the blood samples using the Blood DNA kit (TIANGEN BIOTECH, Beijing, China) and fragmented to 200–300 base pairs using an ultrasonoscope (Covaris S2, Massachusetts, USA). End-repair, adenylation and adapter ligation were performed for library preparation following standard Illumina protocols. Targeted DNA fragments were captured by hybridization to the capture array and sequenced on Illumina HiSeq2000 Analyzers for 90 cycles per read. Image analysis, error estimation and base calling were performed using the Illumina Pipeline (version 1.3.4).

Data analysis and bioinformatics processing were performed as previously described [10]. Reads were aligned to NCBI37/hg19 assembly using the BWA Multi-Vision software package. SNPs and indels were identified using the SOAPsnp software and the GATK Indel Genotyper, respectively. Previously identified SNPs and their allele frequencies were determined using the NCBI dbSNP, 1000 Genomes and the in-house sequencing data of 200 Chinese Han normal hearing controls.

### Confirmation of the potential mutations

Potential mutations detected by targeted NGS were verified by PCR amplification and Sanger sequencing in corresponding probands. For multiplex probands, segregation of the variants with the deaf phenotype was also verified in extended family members when available. The strength of the ectopic splicing sites created by two intronic variants was evaluated by the Human Splicing Finder program (http://www.umd.be/HSF/). Possible pathogenic effects of the missense mutations were evaluated by the Mutation Taster (http://www.mutationtaster.org), PROVEAN and SIFT (with cut-off scores set at −1.3 and 0.05, respectively, http://sift.jcvi.org) programs.

## Results

### Characterization of the deaf probands

A total of 190 probands with non-syndromic deafness was recruited in our study, including 137 simplex and 53 multiplex probands. Seven multiplex probands were from dominant deaf families; four from maternally inherited deaf families; forty-two had at least one first- or second-degree deaf relative and were consistent with a possible recessive inheritance.

Clinical information of the deaf probands was summarized in Table 1. The ages of the probands varied between a few months to over 50 years (mean 14.0 years, 95% CI 11.8-16.3 years). The 7 dominant probands had late-onset, progressive, moderate-to-severe sensorineural hearing impairment. All other probands had prelingual or early-onset, severe-to-profound sensorineural hearing impairment.

**Table 1:** Deaf probands (n=190) categorized by their clinical characteristics

	**Simplex (n=137)**	**Multiplex (n=53)**		
		**Recessive (n=42)**	**Dominant (n=7)**	**Maternal (n=4)**
Sex				
Male	84	19	4	1
Female	53	23	3	3
Age at the test				
0-6 years	44	22	0	0
6-18 years	66	5	1	1
18-35years	23	15	0	3
> 35 years	4	0	6	0
Age of onset				
Prelingual or early onset (≤6 years)	137	42	0	4
Late onset (> 6 years)	0	0	7	0
Severity of hearing impairment				
Mild	0	0	0	0
Moderate	0	0	4	0
Severe	64	15	3	3
Profound	73	27	0	1

### Pre-screening of the common deafness genes

Pre-screening of mutations in three common deafness genes, *GJB2*, *SLC26A4* and *MT-RNR1*, was performed in all 190 deaf probands by Sanger sequencing. Bi-allelic mutations in *GJB2* and *SLC26A4* and the homoplasmic A1555G mutation of *MT-RNR1* were detected in 36, 22 and 7 probands, respectively (Additional file 2: Table S2). Those probands were excluded from further screening by targeted NGS.

### Targeted NGS sequencing of known deafness genes

The remaining 125 deaf probands, including 93 simplex probands, 7 dominant probands, 1 maternally inherited proband and 24 multiplex probands with presumably recessive inheritance went through targeted NGS screening of 79 deafness genes (Additional file 1: Table S1). Included in the list were 50 non-syndromic deafness genes (24 autosomal recessive, 17 autosomal dominant, 6 autosomal recessive/dominant, 2 X-linked and 1 mitochondrial), 7 non-syndromic/syndromic deafness genes (4 autosomal recessive, 2 autosomal recessive/dominant and 1 mitochondrial) and 22 syndromic deafness genes. A total of 1496 exons, 340995 bases (233790 bases in coding regions and 107205 bases in flanking intronic and untranslated regions) were captured and sequenced in our study. After alignment to the reference human genome (NCBI37/hg19), 47.28% of the clean reads could be uniquely matched to the targeted regions. The average depth for the targeted regions was 293.21-fold. 99.35% of the targeted regions were covered by 20 or more reads, demonstrating the high quality of the sequencing.

### Mutation identification and verification

Hundreds of variants of high sequencing quality were detected in each proband. To identify the most likely pathogenic mutations, we filtered out: 1) all previously identified SNPs with allele frequencies of 0.005 or higher, 2) synonymous variants in the coding region and 3) variants in the intronic or untranslated regions (with the exception of the splicing site mutations or variants that may create an ectopic splicing site).

**Figure 1:**
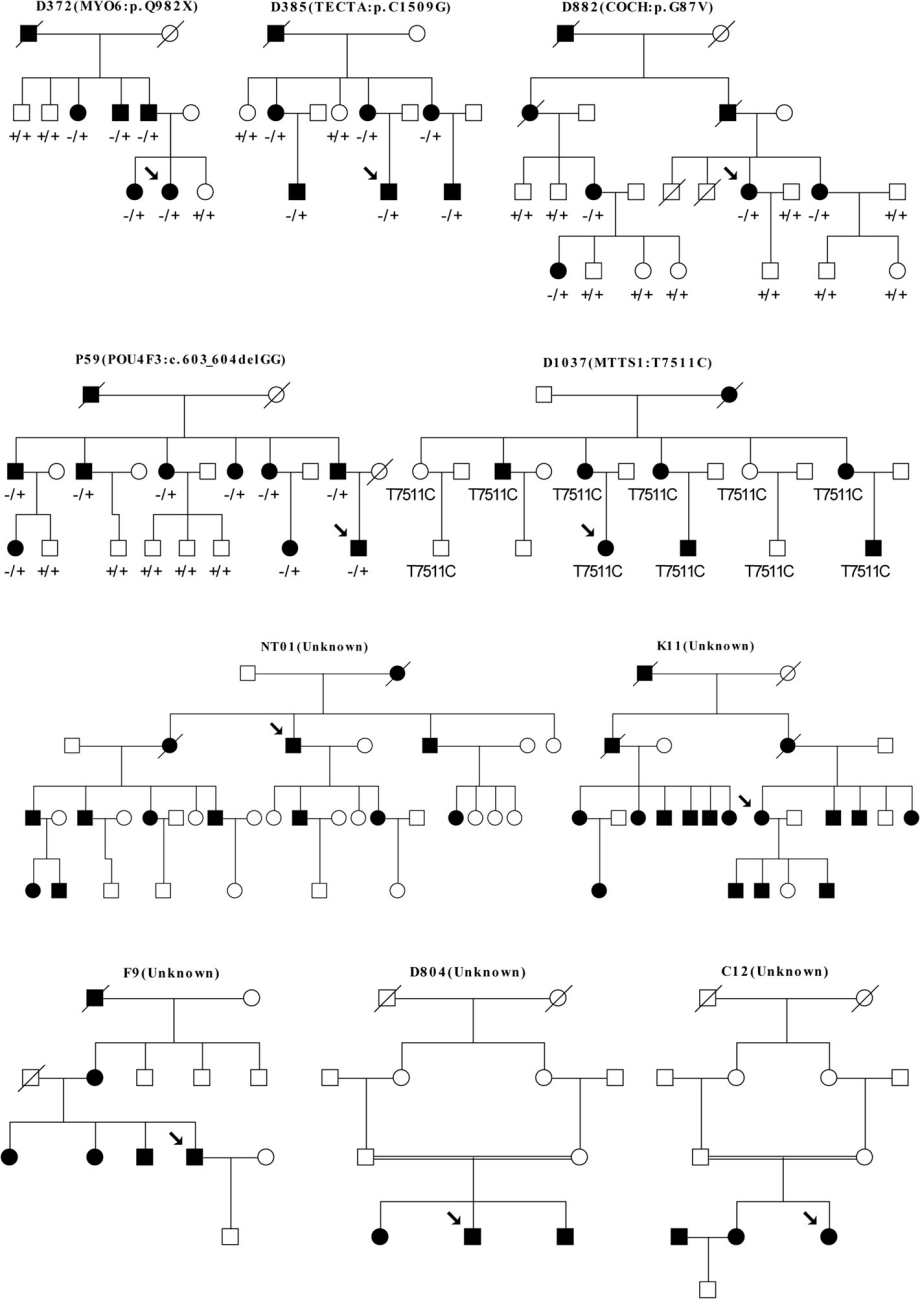
**Pedigrees and the identified mutations of the deaf families (Dominant: D372, D385, D882, P59, NT01, K11 and F9; mitochondrial inherited: D1037; consanguineous: D804 and C12).** Probands of each family were pointed by the arrows.

In 7 dominant probands, we identified 7 heterozygous, non-synonymous variants. None of them was found in 200 ethnically-matched normal hearing controls. Four of them were found co-segregating with the deaf phenotype in extended family members. In addition, a homoplasmic mitochondrial variant was identified in a maternally inherited deaf family with reduced penetrance (Table 2 and Figure 1). Among the five candidate mutations, T7511C in *MT-TS1* and p.C1509G in *TECTA* have been reported to be associated with non-syndromic deafness in previous studies [11-14]. For the other three novel mutations, p.Q982X in *MYO6* and c.603_604delGG in *POU4F3* were predicted to lead to a prematurely stopped protein, while p.G87V in *COCH* changed an evolutionarily conserved amino acid (PhyloP score: 4.75) and was unanimously predicted to be disease-causing by the Mutation Taster, PROVEAN and SIFT programs. Our results suggested that these five mutations in *MYO6*, *TECTA*, *POU4F3*, *COCH* and *MTTS1* were pathogenic.

**Table 2:** Mutations identified in deaf families with dominant or maternal inheritance

**Family ID**	**Gene**	**Type of variation**	**Nucletide change**	**Amino acid change**	**Phylop Score**^**a**^	**Mutation Taster**	**PROVEAN (score)**^**b**^	**SIFT (score)**^**c**^	**Allele frequency in controls**	**Novelty**
D372	*MYO6*	Nonsense	c.2944C>T	p.Q982X	-	DC^d^	-	-	0	Novel
D385	*TECTA*	Missense	c.4525T>G	p.C1509G	4.762	DC	DC (−9.54)	DC (0.004)	0	Reported [12]
D882	*COCH*	Missense	c.260G>T	p.G87V	4.748	DC	DC (−5.63)	DC (0.000)	0	Novel
P59	*POU4F3*	Frame shift	c.603_604delGG	-	-	DC	-	-	0	Novel
D1037	*MTTS1*	Mitochondrial	T7511C	-	-	DC	-	-	0	Reported [13,14]

^a^Score ranges from −14 (not conserved) to 6 (conserved); ^b^Negative and positive scores indicate deleterious and neutral, respectively, with cut-off score set at −1.3; ^c^Score ranges from 0 (deleterious) to 1 (neutral), with cut-off score set at 0.05. ^d^DC: Disease causing.

A total of 254 non-synonymous or splicing site variants were identified in 117 simplex or recessive multiplex probands with allele frequencies of 0.0025 or lower (Additional file 3: Table S3). Consistent with the recessive inheritance, 48 candidate mutations in 15 deafness genes were present in homozygotes (6 probands) or compound heterozygotes (22 probands, Table 3). Three of them, p.R2728H in *MYO15A*, p.R445H and c.150delT in *TMC1*, have been previously reported to be associated with non-syndromic deafness [8,15,16]. Among the other 45 novel candidate mutations, sixteen were nonsense mutations, frame-shifting indels or splicing site mutations. In addition, two intronic variants, c.6956+9C>G in *MYO15A* and c.4961-3C>G in *OTOF*, were predicted to create a strong ectopic splicing site (HSF score of 80.6 and 75.0, respectively) by the Human Splicing Finder program. These 18 mutations were predicted to lead to a significantly truncated protein product. For the remaining 25 missense variants and 2 in-frame deletions, all of them changed evolutionarily conserved amino acids with positive PhyloP scores and were predicted to be disease-causing by at least one of the Mutation Taster, PROVEAN and SIFT programs. Nineteen of 25 missense mutations were unanimously predicted to be disease-causing by all three programs. None of the 48 candidate mutations were found in the 200 Chinese Han normal hearing controls. For 9 multiplex probands (noted with asterisks in Table 3), the bi-allelic mutations were also found in their deaf relatives. Based on these results, the 48 recessive candidate mutations were highly likely to be pathogenic.

**Table 3:** Bi-allelic mutations identified in recessive deaf probands

**Proband ID**	**Gene**	**Type of variation**	**Nucletide change**	**Amino acid change**	**Phylop score**^**a**^	**Mutation Taster**	**PROVEAN (score)**^**b**^	**SIFT (score)**^**c**^	**Allele frequency in controls**	**Novelty**
D691	*MYO15A*	Frame-shift indel	c.6306_6307insG	p.A2104CfsX18	-	DC^c^	-	-	0	Novel
		Missense	c.8183G>A	p.R2728H	3.17	DC	DC(−4.66)	DC (0.008)	0	Reported [8]
D768	*MYO15A*	Splicing site	c.6956+9C>G	-	-	-	-	-	0	Novel
		In-frame indel	c.10251_10253	p.F3420del	-	DC	DC (−10.02)	-	0	Novel
			delCTT							
D856	*MYO15A*	Missense	c.8324G>A	p.R2775H	5.89	DC	DC (−4.56)	DC (0.000)	0	Novel
		Nonsense	c.8767C>T	p.R2923X	1.323	DC	-	-	0	Novel
D37^*^	*MYO15A*	Missense	c.6340G>A	p.V2114M	2.138	DC	DC (−2.70)	DC (0.041)	0	Novel
		Splicing site	c.6956+9C>G	-	-	-	-	-	0	Novel
D465	*MYO15A*	Missense	c.3026C>A	p.P1009H	0.29	PO^d^	DC(−2.47)	DC (0.010)	0	Novel
			c.3026C>A	p.P1009H						
D475	*USH2A*	Missense	c.188G>A	p.R63Q	0.422	PO	DC (−1.30)	PO (0.260)	0	Novel
		Missense	c.8342C>T	p.T2781I	1.606	PO	DC (−3.02)	PO (0.179)	0	Novel
D538	*USH2A*	Missense	c.5017C>T	p.L1673F	2.504	DC	DC(−2.27)	DC (0.014)	0	Novel
		Missense	c.7076T>C	p.L2359S	3.941	PO	DC (−3.57)	DC (0.002)	0	Novel
D773^*^	*USH2A*	Frame-shift indel	c.1992_1993insT	p.K665X	-	DC	-	-	0	Novel
		Splicing site	c.9570+1G>A	-	-	-	-	-	0	Novel
D121	*PCDH15*	Missense	c.1286T>C	p.L429P	4.774	DC	DC (−3.46)	DC (0.019)	0	Novel
			c.1286T>C	p.L429P						
D884^*^	*PCDH15*	Missense	c.3862T>C	p.S1288P	4.99	DC	DC(−2.33)	DC (0.010)	0	Novel
		Missense	c.4118C>T	p.T1373I	4.488	DC	DC (−2.99)	DC (0.002)	0	Novel
D349^*^	*PCDH15*	Frame-shift indel	1438delT	p.S480RfsX1	-	DC	-	-	0	Novel
		Missense	c.4118C>T	p.T1373I	4.488	DC	DC (−2.99)	DC (0.002)	0	Novel
C5	*GPR98*	Frame-shift indel	c.1379delA	p.Q462RfsX36	-	-	-	-	0	Novel
		Frame-shift indel	c.4878_4879ins	p.A1630IfsX1	-	-	-	-	0	Novel
			TTTGCTAATA							
D593^*^	*GPR98*	Missense	c.6559A>G	p.I2187V	0.679	DC	PO (−0.73)	DC (0.041)	0	Novel
			c.6559A>G	p.I2187V						
D29^*^	*GPR98*	Missense	c.2399G>A	p.R800Q	5.497	DC	DC(−2.680)	DC (0.000)	0	Novel
		Frame-shift indel	c.10088_10091	p.V3363DfsX10	-	-	-	-	0	Novel
			delTAAG							
D472	*TMC1*	Splicing site	c.236+1G>C	-	-	-	-	-	0	Novel
		Missense	1334G>A	p.R445H	6.306	DC	DC (−4.97)	DC (0.000)	0	Reported [15]
D419	*TMC1*	Frame-shift indel	c.150delT	p.N50KfsX25	-	-	-	-	0	Reported [16]
		Missense	c.1107C>A	p.N369K	2.211	DC	DC (−5.76)	DC (0.001)	0	Novel
D555	*TMC1*	Missense	c.1209G>C	p.W403C	6.376	DC	DC (−8.97)	DC (0.000)	0	Novel
			c.1209G>C	p.W403C						
D433	*CDH23*	Missense	c.2752G>C	p.D918H	6.036	DC	DC (−5.49)	DC (0.000)	0	Novel
		Missense	c.6606C>A	p.D2202E	3.482	DC	DC (−2.90)	DC (0.013)	0	Novel
D32^*^	*CDH23*	Missense	c.7630T>G	p.L2544V	0.393	DC	PO (−0.54)	PO (0.404)	0	Novel
		Missense	c.8257G>A	p.A2753T	5.768	DC	DC (−1.40)	DC (0.011)	0	Novel
D111	*OTOF*	Nonsense	c.4225A>T	p.K1409X	1.376	DC	-	-	0	Novel
		Splicing site	c.4961-3C>G	-	-	-	-	-	0	Novel
D364	*MYO7A*	In-frame indel	c.1923_1931del	p.KKP642_644del	>1.783	PO	DC (−25.72)	-	0	Novel
			CAAGAAGCC							
		Nonsense	c.6049C>T	p.Q2017X	5.211	DC	-	-	0	Novel
D499	*ESRRB*	Missense	c.1144C>T	p.R382C	6.124	DC	DC (−3.01)	DC (0.039)	0	Novel
		Missense	c.1171T>C	p.L424P	4.995	DC	DC (−6.73)	DC (0.002)	0	Novel
D887	*MARVELD2*	Frame-shift indel	c.1221_1222	p.E408SfsX1	-	DC	-	-	0	Novel
			delAG							
		Splicing site	c.1332-2A>G	-	-	-	-	-	0	Novel
D715	*TECTA*	Nonsense	c.990C>A	p.Y330X	2.723	DC	-	-	0	Novel
			c.990C>A	p.Y330X						
C14	*WHRN*	Missense	c.1292G>A	p.R431Q	4.083	DC	DC (−2.12)	DC (0.010)	0	Novel
		Missense	c.2542A>G	p.R848G	0.487	DC	DC(−1.99)	DC (0.030)	0	Novel
D386	*MYO6*	Missense	c.734A>G	p.Y245C	4.416	DC	DC (−8.77)	DC (0.001)	0	Novel
		Missense	c.1015C>T	p.R339W	1.35	DC	DC (−4.36)	DC (0.003)	0	Novel
D360^*^	*PTPRQ*	Missense	c.5786G>C	p.R1929T	2.965	DC	DC (−2.38)	DC (0.023)	0	Novel
		Nonsense	c.6226C>T	p.Q2076X	5.726	DC	-	-	0	Novel
D366^*^	*POU3F4*	Frame-shift indel	c.644_645insG	p.L217VfsX8	-	DC	-	-	0	Novel
			c.644_645insG	p.L217VfsX8						

^*^Multiplex probands with bi-allelic variants also detected in their deaf relatives; ^a^Score ranges from −14 (not conserved) to 6 (conserved); ^b^Negative and positive scores indicated deleterious and neutral function, respectively, with cut-off score set at −1.3; ^c^Score ranges from 0 (deleterious) to 1 (neutral), with cut-off score set at 0.05; ^c^DC: Disease causing; ^d^PO: Polymorphism.

Overall, our targeted NGS screening identified one mitochondrial mutation in *MTTS1* and four dominant mutations in *MYO6*, *TECTA*, *POU4F3* and *COCH* each. For recessive deafness genes, bi-allelic mutations were identified in 19 simplex and 9 multiplex probands, respectively. Mutations in *MYO15A* were most frequently detected (5/125), followed by mutations in *USH2A*, *PCDH15*, *GPR98*, *TMC1* (3/125 each), *CDH23* (2/125), *OTOF*, *MYO7A*, *ESRRB*, *MARVELD2*, *TECTA*, *WHRN*, *MYO6*, *PTPRQ* and *POU3F4* (1/125 each).

## Discussion

We investigated the genetic epidemiology of non-syndromic deafness in the present study. Like most deaf patients seeking genetic testing and counseling, the majority of our subjects (179/190) were simplex or recessive multiplex probands with non-syndromic, prelingual or early-onset, severe-to-profound hearing impairment. Unlike the syndromic deafness or the progressive, late-onset hearing impairment with representative audioprofiles, this type of hearing impairment is extremely heterogeneous and was often difficult to determine the genetic causes.

We attempted to solve this problem by targeted NGS screening of 79 deafness genes, including all known 57 non-syndromic deafness genes at the time when this study was designed. An additional 22 syndromic deafness genes were also included in the screening in case that any additional symptoms of syndromic deafness were either overlooked or absent during the clinical evaluation (see discussion below on *USH2A* mutations and Usher Syndrome type 2A). We focused on the presumably rare deafness genes by pre-exclusion of mutations in three commonly screened deafness genes *GJB2*, *SLC26A4* and *MT-RNR1*. A strict criterion was applied to identify the possible pathogenic mutations including limiting the candidate mutations only to those with allele frequencies of 0.0025 or lower (based on our in-house sequencing data of 200 ethnically-matched normal hearing controls), recognizing only the bi-allelic variants as candidate recessive mutations, confirming the genotype-phenotype co-segregation with extended family members of the multiplex probands, and using a variety of analysis programs to predict the effects of the candidate mutations.

Consistent with our presumptions of the heterogeneity and the low mutation frequencies of the less commonly screened deafness genes, our targeted NGS screening identified a wide range of dominant, recessive and mitochondrial mutations in 20 deafness genes, whereas no individual gene had mutation detection rate over 3%. Overall, however, those less commonly screened deafness genes contributed to 17.4% (33/190) of causes in our deaf probands, illustrating the importance of genetic testing in those genes.

Several limitations need to be noted when interpreting our results. First, the majority of the probands were recruited through genetic testing and counseling of patients with hereditary hearing loss, which may lead to over-representation of the multiplex probands (27.9% in our subjects, estimated 22.3% in US in comparison[2]). The percentages of the identified genetic causes, therefore, may be slightly over-represented accordingly. Second, our mutation identification criteria selected only the bi-allelic variants as the candidate recessive mutations. It was possible that a small number of probands with mono-allelic mutations may have another undisclosed mutation in the untargeted regions of the genes such as the introns or the regulatory regions, resulting in slight under-representation of the identified genetic causes. Third, mutations in *USH2A* were associated with Usher Syndrome type 2A, a syndromic form of deafness characterized by congenital deafness and later development of progressive retinitis pigmentosa (RP). Because of the late onset of RP (usually postpubertal) in Usher Syndrome type 2A patients, it was not surprising that we identified bi-allelic *USH2A* mutations in three “non-syndromic” deaf probands, all under 6 years old.

**Figure 2:**
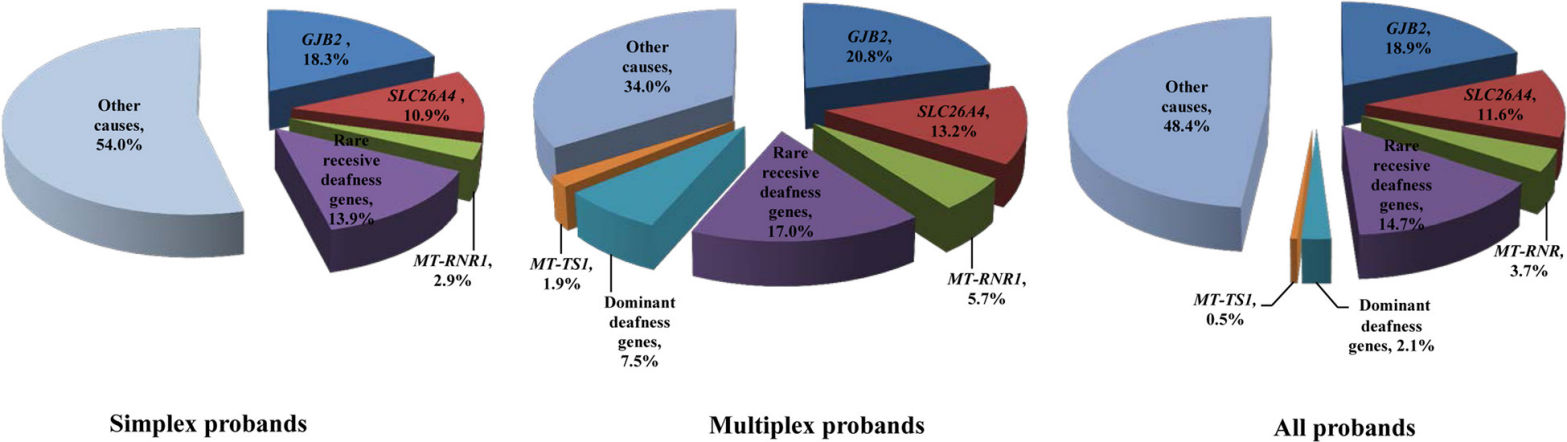
Estimates of genetic causes of non-syndromic deafness in Chinese Hans based on our study.

Nevertheless, our study presented a preliminary overview of the genetic etiology of non-syndromic deafness in Chinese Hans (Figure 2). In addition to *GJB2*, *SLC26A4* and *MT-RNR1*, a variety of less commonly screened deafness genes accounted for a significant portion of genetic causes of non-syndromic deafness. A total of 53 different types of mutations in 20 deafness genes were identified, with 48 of them being novel. Our results confirmed the effectiveness of the targeted NGS screening of the known deafness genes.

Interestingly, our comprehensive screening of 79 deafness genes did not identify any pathogenic mutations in three dominant families and two consanguineous families (Figure 1). We postulated that hearing impairment in these families was likely to be caused by mutations in novel deafness genes and warranted further studies by linkage analysis and/or whole-exome sequencing. Used as a complementary step, this pre-exclusion strategy against known deafness genes may facilitate the discovery of novel deafness genes.

## Abbreviations

NGS: Next-generation sequencing; PTA: Pure-tone audiometry; ABR: Auditory brainstem response (ABR); CT: Computed tomography; RP: Retinitis pigmentosa.

## Competing interests

The authors declare that they have no competing interests.

## Authors’ contributions

TY and HW conceived and designed the experiments. TY, HW, YC and LL ascertained the probands and collected the DNA samples. XW performed the targeted NGS screening and bioinformatics analyses. YC and LL performed the auditory evaluation and other laboratory experiments. TY wrote the paper. All authors read and approved the final manuscript.

## Supplementary Material

Additional file 1: Table S1 Summary of the 79 targeted deafness genes. Click here for file

Additional file 2: Table S2 Mutations in the three commonly screened deafness genes. Click here for file

Additional file 3: Table S3 Variants identified in simplex or recessive multiplex probands. Click here for file
