# Supplementary material for: Genetic etiology study of the non-syndromic deafness in Chinese Hans by targeted next-generation sequencing
Source: Orphanet J Rare Dis. 2013 Jun 14;8:85. doi: 10.1186/1750-1172-8-85 (PMC3703291; doi:10.1186/1750-1172-8-85)
Supplement: Additional file 1: Table S1 — Summary of the 79 targeted deafness genes. [file 1750-1172-8-85-S1.doc]

**Supplementary Table 1**. Summary of the 79 targeted deafness genes.

| **Gene** | **NS/S** | **Inheritance** | **Exons** | **Total length (bases)** | |
| --- | --- | --- | --- | --- | --- |
|  |  |  |  | **Coding** | **Non-coding** |
| *GJB2* | NS | AR/AD | 2 | 681 | 1649 |
| *GJB6* | NS | AR/AD | 3 | 786 | 1285 |
| *MYO7A* | NS/ S | AR/AD | 49 | 6648 | 817 |
| *MYO15A* | NS | AR | 65 | 10593 | 1269 |
| *SLC26A4* | NS/S | AR | 21 | 2343 | 2583 |
| *TMIE* | NS | AR | 4 | 471 | 1390 |
| *TMC1* | NS | AR/AD | 24 | 2283 | 918 |
| *TMPRSS3* | NS | AR | 13 | 1365 | 1076 |
| *OTOF* | NS | AR | 47 | 5994 | 1161 |
| *CDH23* | NS/S | AR | 68 | 10065 | 1044 |
| *STRC* | NS | AR | 29 | 5328 | 186 |
| *USH1C* | NS/S | AR | 27 | 2700 | 546 |
| *TECTA* | NS | AR/AD | 23 | 6468 | 0 |
| *OTOA* | NS | AR | 28 | 3420 | 189 |
| *PCDH15* | NS/S | AR | 33 | 5868 | 1153 |
| *RDX* | NS | AR | 14 | 1752 | 2736 |
| *GRXCR1* | NS | AR | 4 | 873 | 116 |
| *TRIOBP* | NS | AR | 24 | 7098 | 3046 |
| *CLDN14* | NS | AR | 3 | 720 | 1238 |
| *MYO3A* | NS | AR | 35 | 4851 | 933 |
| *WHRN* | NS | AR | 12 | 2724 | 1322 |
| *ESRRB* | NS | AR | 11 | 1527 | 1485 |
| *ESPN* | NS | AR | 13 | 2565 | 965 |
| *MYO6* | NS | AR/AD | 35 | 3858 | 4804 |
| *HGF* | NS | AR | 18 | 2187 | 617 |
| *MARVELD2* | NS | AR | 7 | 1677 | 466 |
| *COL11A2* | NS/S | AR/AD | 63 | 4890 | 1214 |
| *PJVK* | NS | AR | 7 | 1059 | 462 |
| *SLC26A5* | NS | AR | 20 | 2058 | 460 |
| *LRTOMT* | NS | AR | 9 | 756 | 2948 |
| *LHFPL5* | NS | AR | 4 | 660 | 1487 |
| *LOXHD1* | NS | AR | 40 | 6636 | 218 |
| *TPRN* | NS | AR | 4 | 2136 | 505 |
| *GPSM2* | NS | AR | 15 | 2055 | 984 |
| *PTPRQ* | NS | AR | 42 | 6900 | 1166 |
| *GJB3* | NS | AR/AD | 2 | 813 | 1403 |
| *DIAPH1* | NS | AD | 27 | 3792 | 1970 |
| *KCNQ4* | NS | AD | 13 | 1926 | 247 |
| *MYH14* | NS | AD | 42 | 6012 | 801 |
| *DFNA5* | NS | AD | 10 | 1491 | 1004 |
| *WFS1* | NS | AD | 8 | 2673 | 966 |
| *COCH* | NS | AD | 12 | 1653 | 905 |
| *EYA4* | NS | AD | 20 | 1920 | 3772 |
| *POU4F3* | NS | AD | 2 | 1017 | 165 |
| *MYH9* | NS | AD | 41 | 5883 | 1621 |
| *ACTG1* | NS | AD | 6 | 1128 | 791 |
| *SLC17A8* | NS | AD | 12 | 1770 | 2212 |
| *GRHL2* | NS | AD | 16 | 1878 | 3349 |
| *DSPP* | NS | AD | 5 | 3906 | 423 |
| *CCDC50* | NS | AD | 12 | 1449 | 7499 |
| *MYO1A* | NS | AD | 28 | 3132 | 480 |
| *MIRN96* | NS | AD | - | 78 | |
| *TJP2* | NS | AD | 21 | 3132 | 1028 |
| *TIMM8A* | S | X-linked | 2 | 294 | 1148 |
| *PRPS1* | NS | X-linked | 7 | 957 | 1198 |
| *POU3F4* | NS | X-linked | 1 | 1443 | 64 |
| *MTRNR1* | NS | Mitochondrial | - | 954 | |
| *MTTS1* | NS/S | Mitochondrial | - | 69 | |
| *MTTL1* | S | Mitochondrial | - | 75 | |
| *MTTK* | S | Mitochondrial | - | 70 | |
| *MTTE* | S | Mitochondrial | - | 69 | |
| *COL4A5* | S (Alport) | X-Linked | 51 | 5058 | 1369 |
| *EYA1* | S (BOR) | AD | 18 | 1779 | 2545 |
| *SIX1* | S (BOR) | AD | 2 | 855 | 1831 |
| *SIX5* | S (BOR) | AD | 3 | 2220 | 1110 |
| *KCNE1* | S (JLN) | AR | 3 | 390 | 2930 |
| *KCNQ1* | S (JLN) | AR | 16 | 2031 | 1214 |
| *COL11A1* | S (STL) | AD | 66 | 5073 | 2100 |
| *TCOF1* | S (TCO) | AD | 26 | 4236 | 628 |
| *USH1G* | S (USH) | AR | 3 | 1386 | 2175 |
| *USH2A* | S (USH) | AR | 72 | 15609 | 3274 |
| *CLRN1* | S (USH) | AR | 3 | 699 | 1660 |
| *GPR98* | S (USH) | AR | 90 | 18921 | 411 |
| *MITF* | S (WS) | AD | 10 | 1563 | 3203 |
| *PAX3* | S (WS) | AD | 9 | 1452 | 1886 |
| *SNAI2* | S (WS) | AR | 3 | 807 | 1291 |
| *EDNRB* | S (WS) | AR/AD | 8 | 1329 | 2953 |
| *EDN3* | S (WS) | AR/AD | 6 | 717 | 1680 |
| *SOX10* | S (WS) | AD | 4 | 1401 | 1461 |
| Total | - | - | 1496 | 233790 | 107205 |

NS: Non-Syndromic; S: Syndromic; AR: Autosomal Recessive; AD: Autosomal Dominant; BOR: Branchio-oto-renal syndrome; JLN: Jervell & Lange-Nielsen syndrome; STL: Stickler syndrome; TCO: Treacher Collins syndrome; USH: Usher syndrome; WS: Waardenburg syndrome.
