# Supplementary material for: Genetic etiology study of the non-syndromic deafness in Chinese Hans by targeted next-generation sequencing
Source: Orphanet J Rare Dis. 2013 Jun 14;8:85. doi: 10.1186/1750-1172-8-85 (PMC3703291; doi:10.1186/1750-1172-8-85)
Supplement: Additional file 2: Table S2 — Mutations in the three commonly screened deafness genes. [file 1750-1172-8-85-S2.doc]

**Supplementary Table 2.** Mutations in the three commonly screened deafness genes.

| **Bi-allelic mutations** | **Number (%) of probands** | |
| --- | --- | --- |
|  | **Simplex (n=137)** | **Multiplex (n=53)** |
| *GJB2* | 25(18.25) | 11(20.75) |
| c.235delC/c.235delC | 9(6.57) | 5(9.43) |
| c.235delC/ c.299-300delAT | 5(3.65) | 3(5.66) |
| c.235delC/c.176-191del16 | 3(2.19) | 2(3.77) |
| c.235delC/c.507insAACG | 1(0.73) | 1(1.89) |
| c.176-191del16/ c.299-300delAT | 1(0.73) | - |
| c.235delC/p.T86R | 1(0.73) | - |
| *SLC26A4* | 15(10.95) | 7(13.21) |
| c.919-2A>G/ c.919-2A>G | 6(4.38) | 4(7.55) |
| c.919-2A>G/ p.H723R | 2(1.46) | 1(1.89) |
| c.919-2A>G / p.R409H | 2(1.46) | 1(1.89) |
| c.919-2A>G/c.600+2T>A | 1(0.73) | - |
| c.919-2A>G/ p.M147V | 1(0.73) | - |
| c.919-2A>G/ p.V659L | 1(0.73) | - |
| p.T410M/ c.1614+1G>A | 1(0.73) | - |
| p.R409H/ p.H723R | 1(0.73) | - |
| p.N392Y/p.H723R | - | 1(1.89) |
| *MT-RNR1* | 4(2.92) | 3(5.66) |
| A1555G | 4(2.92) | 3(5.66) |
| Total | 44(32.12) | 21(39.62) |
