# Supplementary material for: Genetic etiology study of the non-syndromic deafness in Chinese Hans by targeted next-generation sequencing
Source: Orphanet J Rare Dis. 2013 Jun 14;8:85. doi: 10.1186/1750-1172-8-85 (PMC3703291; doi:10.1186/1750-1172-8-85)
Supplement: Additional file 3: Table S3 — Variants identified in simplex or recessive multiplex probands. [file 1750-1172-8-85-S3.doc]

**Supplementary Table 3.** Variants identified in simplex or recessive multiplex probands.

| **Gene** | **cDNA** | **No. of variants** | **Variants** |
| --- | --- | --- | --- |
| *GJB6* | NM_006783 | 2 | p.V23A; p.P232L; |
| *MYO7A* | NM_000260 | 15 | p.D2010N; p.K2118Q; p.V1669I; p.G958D; **c.1923_1931delCAAGAAGCC, p.Q2017X**; p.R2070Q; p.A1437T; p.T1748M; p.R1526H; p.V1669I; p.E1100Q; p.P541R; p.F614L; (p.L1484I) |
| *MYO15A* | NM_016239 | 23 | **p.V2114M, c.6956+9C>G;** p.R1107H; (p.E3305K); p.R3389W; p.P813S; p.G51D; p.V2872M; **p.G2231A, c.7787+4A>G;** **p.P1009H, p.P1009H**; p.S2426G; **c.6306_6307insG, p.R2728H**; (p.R1754H); p.D2545Y; **c.6956+9C>G, c.10251_10253delCTT**; **p.R2775H, p.R2923X**; **c.5964+3G>A, c.8791delT**; |
| *SLC26A4* | NM_000441 | 7 | p.K44N; p.I300T; p.K44N; p.N382K; p.R409H; (c.600+2T>A); p.R409H; |
| *TMC1* | NM_138691 | 7 | **c.150delT, p.N369K**; p.S697X; **c.236+1G>C, p.R445H**; **p.W403C, p.W403C**; |
| *OTOF* | NM_194248 | 7 | p.R518L; **p.K1409X, c.4961-3C>G**; p.I874F; (p.I101T); (p.N743D); (p.N743D); |
| *CDH23* | NM_022124 | 13 | **p.L2544V, p.A2753T**; (p.V1908I); p.P888L; p.R736Q; p.L1614P; **p.N918H, p.D2202E**; p.L693P; p.S2462P; p.R1189W; (p.G1118S); p.T980M; |
| *USH1C* | NM_005709 | 2 | p.P176T; (p.D265N); |
| *TECTA* | NM_005422 | 5 | p.R1477C; p.H1377L; **p.Y330X, p.Y330X**; p.M117V; |
| *OTOA* | NM_144672 | 2 | p.E144D; p.I140L; |
| *PCDH15* | NM_033056 | 12 | p.K366X; (p.S399R); p.D984Va; **p.L429P, p.L429P**; c.3717+1G>A; p.A1376D; **1438delT, p.T1373I**; p.R1350H; **p.S1288P, p.T1373I**; |
| *GRXCR1* | NM_001080476 | 1 | p.N280S |
| *TRIOBP* | NM_001039141 | 8 | p.Q1337E; p.C772R; p.Q1972R; p.R2131Q; p.A531T; F1174S; (p.A531T); c.5185-2A>G; |
| *CLDN14* | NM_001146077 | 1 | p.A216T; |
| *MYO3A* | NM_017433 | 1 | p.Q802K |
| *WHRN* | NM_015404 | 6 | **p.R431Q, p.R848G**; p.R490H; p.M503L; p.R837C; p.A458V; |
| *ESRRB* | NM_004452 | 2 | **p.R382C, p.L424P**; |
| *ESPN* | NM_031475 | 4 | p.G423D; p.Q8E; p.T742P; p.E776K; |
| *MYO6* | NM_004999 | 4 | c.3842_3843delAG; **p.Y245C, p.R339W**; p.R1010Q; |
| *MARVELD2* | NM_001038603 | 4 | (p.R372Q); p.M490I; **c.1221_1222delAG, c.1332-2A>G**; |
| *COL11A2* | NM_080679 | 1 | p.M1480V; |
| *LRTOMT* | NM_001145310 | 2 | p.R127H; p.M1K; |
| *LOXHD1* | NM_144612 | 11 | p.R587W; p.I1515V; (p.R615Q); (p.D1094G); p.A679T; p.V1959I; c.5888delG; p.R1124H; p.R2143C; p.L1496F; (p.E1917K); |
| *TPRN* | NM_001128228 | 1 | p.H664R; |
| *PTPRQ* | NM_001145026 | 9 | p.E1165D; p.G1905S; p.T220A; p.R1987Q; **p.R1929T, p.Q2076X**; c. 4382_4384delTTG; p.D1471G; p.R1298H |
| *GJB3* | NM_024009 | 2 | p.R180L; p.R101W; |
| *DIAPH1* | NM_001079812 | 5 | p.Y415C; (p.I691N); p.D68G; p.R959H; p.L778P; |
| *KCNQ4* | NM_004700 | 2 | p.R689Q; p.P263L; |
| *MYH14* | NM_001077186 | 6 | p.K621N; p.Y434C; p.R336W; p.A990V; p.Q25K; p.Q348R; |
| *DFNA5* | NM_004403 | 1 | p.E81K |
| *WFS1* | NM_001145853 | 5 | p.A874T; p.A179T; p.A58V; p.R791C; p.S790L; |
| *COCH* | NM_001135058 | 1 | p.G75R |
| *EYA4* | NM_172105 | 1 | p.Q233R |
| *MYH9* | NM_002473 | 3 | p.A1469V; p.R1605C; p.S1771T; |
| *ACTG1* | NM_001614 | 1 | p.S300W |
| *GRHL2* | NM_024915 | 4 | (p.A28G); p.T343M; p.C288Y; p.I186M; |
| *DSPP* | NM_014208 | 4 | (p.D1152N); p.D1035N; (p.D1254N); p.D943E; |
| *CCDC50* | NM_178335 | 2 | p.T229A; p.R103H |
| *MYO1A* | NM_005379 | 2 | p.D221Y; p.N27S; |
| *TJP2* | NM_001170414 | 4 | p.D507G; c.2897+3A>G; p.Q733R; p.V498M; |
| *POU3F4* | NM_000307 | 2 | **c.644_645insG, c.644_645insG** |
| *COL4A5* | NM_000495 | 2 | p.Q1383K; p.P1546T |
| *SIX1* | NM_005982 | 1 | p.V106M |
| *SIX5* | NM_175875 | 2 | p.V551M; p.L671P; |
| *KCNE1* | NM_000219 | 1 | p.Q96X |
| *KCNQ1* | NM_000218 | 1 | p.R192C; |
| *COL11A1* | NM_080630 | 3 | (p.P912L); p.A147S; (p.T187M); |
| *TCOF1* | NM_001135243 | 1 | (p.T316A); |
| *USH1G* | NM_173477 | 4 | p.G208S; p.K130T; **c.164+5G>A, c.164+5G>A**; |
| *SH2A* | NM_206933 | 22 | p.R1870W; (p.I3082V); p.S4881T; p.H2346R; p.R3941W; **p.R63Q, p.T2781I**; p.D3463G; **p.L1673F, p.L2359S**; p.P2533R; **p.P392S, p.E2613D**; p.G1708R; **c.1992_1993insT, c.9570+1G>A**; p.T3015A; (p.E4264K); c.6325+1G>A; p.G2017D; **c.6485+5G>A, c.8559-2A>G**; |
| *GPR98* | NM_032119 | 20 | **c.1379delA, c.4878_4879insTTTGCTAATA**; p.D5881Y; (p.E1391K); **c.10088_10091delTAAG, p.R800Q**; p.Y6058C; p.T4000M; p.A6191P; p.A5957G; p.Q5861H; p.I4484T; p.Y305H; p.P4155S; **p.I2187V, p.I2187V**; p.H4426R; p.D3992N; p.G6285R; p.T5725A |
| *PAX3* | NM_181459 | 1 | p.T31S |
| *EDN3* | NM_000114 | 1 | p.T185M; |
| *Total* |  | 254 |  |

Bi-allelic variants were in bold; variants identified once in 200 normal hearing controls (allele frequencies of 0.0025) were in parentheses
